# Clinical and Dermoscopic Findings of Nevi after Photoepilation: A Review

**DOI:** 10.3390/life13091832

**Published:** 2023-08-29

**Authors:** Clio Dessinioti, Andriani Tsiakou, Athina Christodoulou, Alexander J. Stratigos

**Affiliations:** 1 1st Department of Dermatology-Venereology, Andreas Sygros Hospital, National and Kapodistrian University of Athens, 16121 Athens, Greece; 2 State Department of Dermatology-Venereology, Andreas Sygros Hospital, 16121 Athens, Greece

**Keywords:** melanocytic, nevi, laser, hair removal, IPL, changes, dermoscopy

## Abstract

Atypical clinical and dermoscopic findings, or changes in pigmented melanocytic lesions located on body areas treated with lasers or intense pulsed light (IPL) for hair removal (photoepilation), have been described in the literature. There are three prospective studies in a total of 79 individuals with 287 melanocytic nevi and several case reports reporting the dermoscopic findings and changes after photoepilation. Clinical changes have been reported in 20–100% of individuals, while dermoscopic changes have been observed in 48% to 93% of nevi. More frequent dermoscopic changes included bleaching, the development of pigmented globules, and irregular hyperpigmented areas and regression structures, including gray areas, gray dots/globules, and whitish structureless areas. The diagnostic approach for pigmented lesions with atypical dermoscopic findings and changes after photo-epilation included reflectance confocal microscopy, sequential digital dermoscopy follow-up, and/or excision and histopathology. Challenges pertaining to these diagnostic steps in the context of photoepilation include the detection of findings that may warrant a biopsy to exclude melanoma (ugly duckling, irregular hyperpigmented areas, blue-gray or white areas, and loss of pigment network), the potential persistence of changes at follow-up, and that a histopathologic diagnosis may not be possible due to the distortion of melanocytes or complete regression of the lesion. Furthermore, these diagnostic approaches can be time-consuming, require familiarization of the physician with dermoscopic features, may cause anxiety to the individual, and highlight that avoiding passes of the laser or IPL devices over pigmented lesions is key.

## 1. Introduction

The evaluation of melanocytic lesions for new or changing findings is among the good practices for the identification or exclusion of cutaneous melanoma in adults [1]. In addition, the rationale for the monitoring of new or changed melanocytic nevi is based on the very low possibility that an acquired melanocytic nevus (termed nevus thereafter) may, in some cases, act as a precursor lesion evolving into cutaneous nevus-associated melanoma [2,3,4,5,6,7,8]. Secondary prevention is an important pillar of Dermato-oncology focusing on the education of the public regarding when to seek a physician skin examination and on the training of health care professionals in the best practices to screen melanocytic lesions and detect melanoma [9]. The methods of examination of melanocytic lesions include clinical naked-eye examination, hand-held dermoscopy, total body photography, sequential digital dermoscopy monitoring, reflectance confocal microscopy, and, after biopsy or excision, histopathology. Dermoscopy (epiluminescence microscopy) is a non-invasive in vivo imaging technique that improves the diagnostic accuracy for melanocytic lesions compared to naked-eye examination, and is an established tool incorporated in clinical practice [10,11,12,13].

A nevus consists of melanocytes and melanin, and photons of the lasers used for hair removal might be absorbed by the melanocytes of the nevus and cause subsequent thermal injury [14,15]. Photoepilation is widely used for hair removal and involves the use of lasers (long-pulsed alexandrite laser (755 nm), diode laser (800–810 nm), neodymium:yttrium-aluminum-garnet (Nd:YAG) laser (1064 nm), and ruby laser (694 nm)) or intense pulsed light (IPL) sources [15,16]. Atypical findings have been noted in pigmented lesions after laser or IPL hair removal (named photoepilation hereafter), either as new findings or as changed lesions. Such findings prompt several diagnostic approaches, including dermoscopy and histopathology, in order to establish an unequivocal diagnosis.

We performed a comprehensive literature review, aiming to detail the reported clinical, dermoscopic, and histological findings in pigmented lesions located on areas treated with photo-epilation. To our knowledge, currently there is no review on the morphological, dermoscopic, and histological findings in pigmented lesions after laser or IPL for hair removal. We performed searches in PubMed using the terms “nevi”, “melanocytic”, “pigmented”, “laser” and “hair removal”, “IPL”, “dermoscopic”, “changes”, and “photoepilation”. Also, we carried out secondary referencing by manually reviewing the reference lists of assessed articles.

The results of our literature search are classified into three types of evidence, which are discussed in the following sections. First, the clinical and dermoscopic findings noted in pigmented lesions after photoepilation are detailed. Second, longer-term dermoscopic findings are reported in pigmented lesions with atypical findings that were followed up. Third, in some persons presenting with atypical pigmented lesions after photoepilation, a surgical excision was performed and the histological findings were described. The features of pigmented lesions after photo-epilation in patients with atypical nevi or a history of melanoma are also highlighted.

## 2. Clinical Findings of Pigmented Lesions after Laser or IPL Hair Removal 

Clinical morphological changes in nevi in the field of laser treatment for hair removal, are inflammatory reactions ranging from swelling and/or redness to oozing, ulceration, crust, and variable loss of color or regression of the nevi [17,18]. Clinical changes have been noted in 20% [17] to 59% [18] of individuals and in 20.2% [18] to 100% [19] of nevi, depending on the timing of follow-up in relation to the sessions of photoepilation. In the prospective study of Acle et al., in 34 women with 148 nevi on the legs, who underwent six sessions of hair removal with a diode laser, clinical changes were noted in 20% of nevi on the legs compared to 0 in control nevi (on arms). The clinical changes included bleaching in 20% and partial regression in 3% [18]. In the prospective study of Guicciardi et al., in 18 persons with 73 melanocytic lesions who had photo-epilation and were monitored for at least 2 years, changes in color were observed in all nevi, which became clearer but also had an irregular distribution of pigmentation [19].

Clinical atypical findings in pigmented lesions after photoepilation may prompt a biopsy or surgical excision so that the histopathologic assessment will contribute to diagnosis. A nevus that was darkly pigmented and symmetrical, with a typical pigment network, but that was different from the patient’s signature nevus and darker and larger than other nevi, was noted on the back of an individual after two sessions of diode laser hair removal. The nevus was biopsied and subsequently excised [20]. Similarly, in another case, clinical changes were noted by a woman with a family history of melanoma, after diode laser epilation. The nevus was clinically darker and different in dermoscopy from the other nevi, and it was excised [21].

The concept of “ugly duckling” has been used when a single melanocytic lesion appears to be atypical or shows changes making it obviously different from the individual’s other nevi, and it constitutes a major indicator for suspicion of melanoma [22,23].

In other reports, clinical changes with asymmetry, irregular borders, or color changes prompted the biopsy or excision of the nevi [24]. Clinical changes in pigmented lesions in adults are an important criterion that may signify melanoma, and the letter “E” for evolution is included in the ABCDE mnemonic (asymmetry, border irregularity, color variegation, diameter >6 mm, and evolution), which has long been used in public health messaging to assist in visually detecting melanoma [25,26].

Although the clinical and dermoscopic changes occurring in nevi in the setting of photo-epilation could be attributed to the photo-epilation per se, this is not a certain scenario and a careful evaluation to establish the unequivocal diagnosis of a benign nevus is necessary.

## 3. Dermoscopic Findings of Pigmented Lesions after Laser or IPL Hair Removal

There are three prospective studies in a total of 79 individuals [18,19,27] and several case reports [14,20,21,24,28,29,30,31] reporting atypical dermoscopic findings and changes in pigmented lesions after photo-epilation (Table 1). These findings were confined to the nevi located on body areas that underwent photoepilation, and they were not observed in nevi on areas not treated with photoepilation [28,29,30,31]. There are only limited reports of changes in pigmented lesions after photoepilation in patients with atypical nevi or a history of melanoma, and an excision [21,24,28,29,30,31] or biopsy [24] of the lesion was performed in all these cases, except in one patient who was followed up and in whom dermoscopic changes were resolved [14].

**Figure 1:**
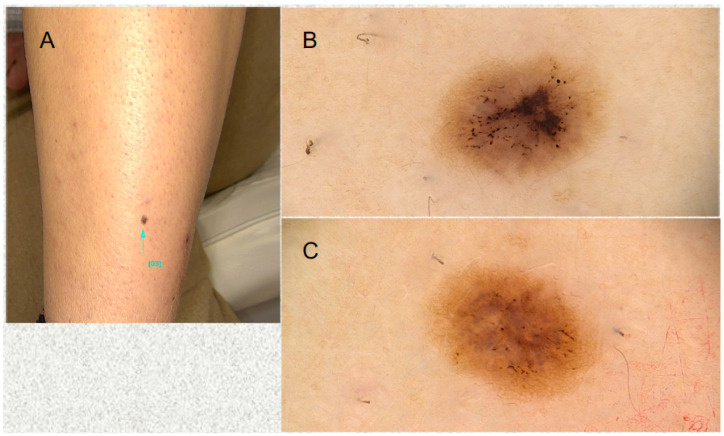
An atypical pigmented lesion was noted on the front of the left lower leg in a 36-year-old woman. She mentioned she had undergone laser hair removal on her legs 3 weeks before. (**A**) Clinical aspect. (**B**) Dermoscopy showing loss of the pigment network, a central dark brownish blotch, and a gray structureless area. (**C**) At 2-week follow-up (and 5 weeks after the laser hair removal), a reticular pigment network is starting to become evident at the periphery. Gray area is still seen in the center of the lesion.

**Figure 2:**
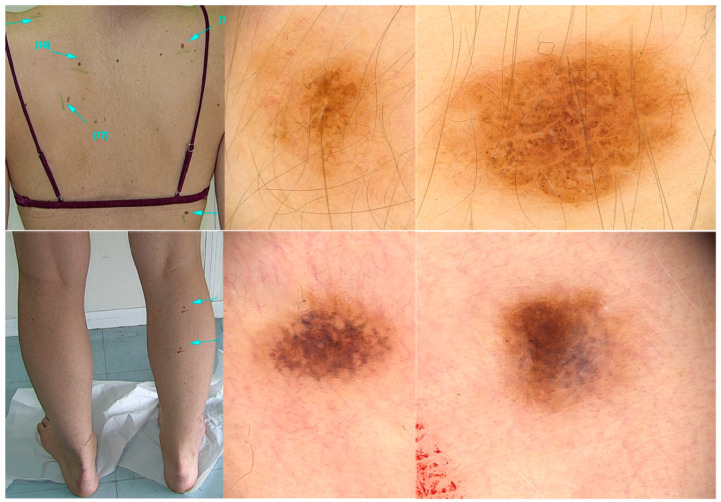
The same woman in Figure 1 at her follow-up visit. (**Upper panel**): Nevi on her back that had not undergone laser hair removal showing a globular pigment network. (**Lower panel**): Lesions on the right calf that had undergone laser hair removal showing persistent changes 5 weeks after photoepilation. In dermoscopy, there is loss of the pigment network and loss of pigmentation particularly evident in one half of the nevus. Grayish areas are also observed in one half of the nevus.

The more frequently reported dermoscopic features after photoepilation in the available prospective studies were bleaching, changes in color, crusting, changes in the pigment network pattern, and regression, including gray areas, gray dots/globules, and whitish structureless areas (Figure 1 and Figure 2) [18,19,27].

The only controlled study was performed by Acle et al. in 34 women with 148 nevi on the legs that underwent six sessions of hair removal with a diode laser; nevi on their arms were used as controls [18]. The use of a control group of nevi on a body area untreated with photoepilation is important to account, at least to some degree, for changes in nevi due to sun exposure or inherent volatility [18]. Before session 6, dermoscopic changes were noted significantly more frequently in nevi on the legs after diode laser hair removal (47.9%) compared to controls (9.8%) (*p* < 0.001). There was an 8-fold higher risk of dermoscopic changes on nevi on the legs after photoepilation compared to nevi that did not undergo photoepilation. Statistically significant dermoscopic changes occurring in nevi of the legs included bleaching in 41.9% (versus 0 in control nevi, *p* < 0.05), pigmented globules in 6.7% (versus 0 in control nevi, *p* < 0.05), irregular hyperpigmented areas in 5.4% (versus 0 in control nevi, *p*: 0.011), and regression structures in 4.7% (versus 0 in control nevi, *p*: 0.021) [18]. Notably, irregular hyperpigmented areas as described in nevi after laser hair removal have also been found to be among the most frequent dermoscopic criteria for the diagnosis of melanoma in situ [32]. These findings highlight that dermoscopic features detected in melanocytic lesions after photoepilation may cause a diagnostic challenge and require further evaluations to reach diagnosis.

Dermoscopic changes in nevi after photoepilation depend on the timing of their assessment and may become more prominent over the sessions of photoepilation. The study of Acle et al. collected the changes occurring in nevi at the second, third, and sixth session performed every 4–7 weeks, compared to before starting photo-epilation. Bleaching was observed in 14.8% before the second session, in 23.6% before third session, and in 41.9% before the sixth session. There was no crusting noted, probably because the nevi were evaluated several weeks after the preceding laser session [18] (Table 1).

Nassimi et al., in a prospective study, reported dermoscopic findings in 66 junctional melanocytic nevi in 27 women, before and 2 months after a single session of alexandrite laser for hair removal. With digital dermoscopy, they noticed a change in color in 64% of nevi, of which 44% became lighter and the remaining 20% became darker, changes in the reticular pattern in 92.5%, changes in the pattern of dots and globules in 71.2% of nevi, and regression in two nevi [27]. Guicciardi et al. performed a prospective study in 73 nevi of 18 patients that underwent hair removal with Nd:Yag laser or IPL and were monitored for at least 2 years. Crusting due to acute burn was reported in 11% of nevi. Dermoscopic changes included bleaching (82%), irregular enhanced pigment network at the periphery (79%), blue-gray globules (46.5%), white structureless areas (37%), and complete regression (32.8%). Only one nevus was excised due to growth and the appearance of peripheral globules, and the histopathology showed a compound nevus with slight atypia [19].

Blue-gray-white areas [28,30,31] and grayish areas [14] after photo-epilation have also been described in case reports. Regarding the significance of blue and white areas in dermoscopy outside the context of photoepilation, they are included in the definition of regression structures and may be detected in benign nevi and in melanoma [33,34,35,36]. Blue areas, synonymously named gray-blue areas, have been defined as small diffuse or speckled zones with a gray-blue or gray hue [33]. Blue color has been correlated histopathologically with melanin within melanophages or pigmented melanocytes in the dermis. White color has been correlated with areas of regression with fibroplasia [33,34,37]. Blue structureless areas were significantly more frequent in equivocal lesions that were excised and diagnosed as nevi and in invasive melanomas compared to melanoma in situ in the study of Seidenari et al. [35]. In that study, blue areas were variably combined with other dermoscopic features of regression, including white areas, peppering, and a blue-whitish veil. Nevi were characterized by the presence of blue areas only, not associated with other regression features. In particular, nevi showed blue areas alone in the majority of cases (76.6%), while melanomas in situ had blue areas alone in 39.4%, and invasive melanomas showed blue areas alone in only 15.2% [35]. In the study of Nazzaro et al., the blue-white veil was a dermoscopic feature associated with the diagnosis of mini-melanomas with diameters ≤5 mm compared to clinically equivocal melanocytic nevi with diameters ≤5 mm. Also, they reported that the blue-white veil was detected more frequently in invasive mini-melanomas compared to in situ mini-melanomas [38]. In addition, reflectance confocal microscopy has recognized plump cells corresponding to melanophages and inflammatory infiltrates in histology within the blue areas recognized by dermoscopy [36]. Furthermore, it has been proposed that the presence of extensive regression is an indication for biopsy even in the absence of dermoscopic criteria for melanoma [39]. According to the algorithm of Zalaudek et al., blue-white structures were defined as white areas, blue areas, or a combination of both (including the blue-white veil), and a biopsy was proposed when blue-white structures occupy over 50% of the lesion [40].

A structureless pattern was observed in a pigmented lesion after photo-epilation in a woman with a family history of melanoma, which was excised [21]. A featureless pattern is characterized by the absence of features specific to a melanocytic lesion. Before the implementation of reflectance confocal microscopy, the management of featureless or feature-poor lesions was to biopsy them or perform sequential digital dermoscopy imaging and then biopsy if further changes occurred [1,41,42]. A “structureless” lesion is considered as a melanocytic lesion with a suspicion of melanoma in the two-step algorithm for the classification of pigmented lesions of the skin. The two-step algorithm is a decision-making method which classifies pigmented lesions according to dermoscopic structures in seven levels [43,44]. In the first step, seven different levels of criteria are used to differentiate melanocytic from nonmelanocytic skin lesions. Lesions that fail to be classified in levels 1 through 6 (e.g., melanocytic, BCC, seborrheic keratoses, vascular lesions, nonmelanocytic lesions with specific blood vessels, or melanocytic lesions with specific blood vessels) are classified as “structureless” in level 7, and they are considered as suspicious for melanoma. This level was introduced to ensure that a melanoma without any detectable structures would not be missed. In the second step, lesions that are diagnosed as melanocytic are further classified as benign melanocytic nevus or melanoma. It was proposed that the differential diagnosis for all structureless lesions should include melanoma and that it should be ruled out via biopsy or short-term dermoscopic imaging [43].

In the literature, the diagnostic approach for pigmented lesions with atypical dermoscopic findings and changes after photoepilation included reflectance confocal microscopy, sequential digital dermoscopy follow-up, and/or excision (Table 2). Reflectance confocal microscopy, when available, is a non-invasive imaging method that allows the cellular assessment of the epidermis and superficial dermis at a resolution approaching histological detail [1,45,46]. Reflectance confocal microscopy has been reported in only two patients with a history of atypical nevus syndrome and atypical dermoscopic findings after photoepilation. There were no atypical cells with reflectance confocal microscopy in either patient, and subsequent excision confirmed the diagnosis of nevi without cellular atypia [30]. Short-term sequential digital dermoscopy may be considered to follow up atypical or changed pigmented lesions to assess for their evolution and resolution. However, these changes can be persistent in some cases, which limits the usefulness of short-term sequential digital dermoscopy and may prompt a biopsy or surgical excision. The dermoscopic findings in pigmented lesions that were followed up and the histological features of lesions that were excised are discussed in the following sections.

## 4. Follow-Up Dermoscopic Findings in Atypical or Changed Pigmented Lesions after Laser or IPL Hair Removal

Sequential digital dermoscopy has been used when a follow-up of the lesion was decided. The follow-up information in pigmented lesions that showed atypical morphological and dermoscopic changes after photoepilation but were not excised and were re-evaluated at a later time is summarized in Table 3. Follow-up has been reported in 60 persons and ranged from 3 to 36 months. The majority of cases had persistent clinical and dermoscopic changes after laser or IPL hair removal, underscoring some limitations and the possible need for longer follow-up times.

A longer follow-up of at least 2 years was provided in the prospective study of Guicciardi et al. Some persons were followed for 6–8 years. The dermoscopic modification was persistent and stable in all subsequent follow-ups, despite stopping photoepilation. The authors mentioned that the stability of features suggested a benign behavior. Changes were suggestive of possible malignant transformation in only one case with progressive growth and peripheral globules that was excised, and histology showed a compound nevus with slight atypia [19]. A longer follow-up of the two cases reported by Garido-Rios et al., three years later, reported that the changes in nevi persisted, were more prominent, and showed loss of pigmentation and of the pigment network despite stopping photoepilation. On the other hand, nevi located in body areas that did not receive laser or IPL hair removal did not show these changes [28].

## 5. Histological Findings in Atypical Pigmented Lesions after Laser or IPL Hair Removal That Were Surgically Excised

The histological findings in excised pigmented lesions after photo-epilation were reported in eleven case reports [14,20,21,24,28,29,30,31] and in one case included in a prospective study [19]. The histological findings and the reasons for surgical excision are shown in Table 4. The histopathological diagnosis of a melanocytic nevus was established in nine persons, while a diagnosis was not possible in three cases [20,21,29]. The latter was due to marked distortion of melanocytes [20] or the complete regression of the lesion [21,29].

Lesions with blotches of brown pigment with no melanocytic pattern with dermoscopy have had small superficial microcrusts in histology. Lesions with central whitish areas with dermoscopy have had fibrosis in the papillary dermis. The complete regression of the pre-existing melanocytic nevus was confirmed with Melan-A immunostaining [29]. Grayish areas with dermoscopy have frequently shown melanophages in histology [14,28,30,31].

It has been suggested that histologic changes reflecting the thermal destruction of the melanocytes, nevus cells, surrounding keratinocytes, and stromal matrix include subepidermal blister formation, melanocytes with marked distortion in their shape or fragmented within the epidermis or the dermal–epidermal junction, and collagen homogenization in the papillary dermis [24].

## 6. Conclusions

Our review summarizes the reported clinical, dermoscopic, and histological findings and changes in pigmented melanocytic lesions after treatment with a laser or IPL for hair removal. The observation that changes were noted in nevi located on body areas that underwent photoepilation [28,29,30,31], as well as the significantly more frequent dermoscopic changes in nevi on areas treated with photoepilation compared to nevi on untreated areas [18], support the potential of these light sources to cause changes in nevi after hair removal. We did not find any report of melanoma diagnosed in the cases with changes in nevi after laser or IPL hair removal in the published literature.

When clinical and dermoscopic changes or atypical findings are noted in a pigmented lesion after photoepilation, an unequivocal diagnosis must be made. Establishing the diagnosis of a nevus and excluding any possibility the lesion was in fact a melanoma entail the diagnostic approach followed in any case of an atypical melanocytic lesion, e.g., a careful dermoscopic evaluation, reflection confocal microscopy (if available), possible dermoscopic follow-up, or surgical excision and histopathology. Challenges pertaining to these diagnostic steps in the context of photoepilation include the detection of findings that may warrant a biopsy to exclude melanoma (ugly duckling, irregular hyperpigmented areas, blue-gray or white areas, and loss of pigment network), the potential persistence of changes at follow-up, and that a histopathologic diagnosis may not be possible due to the distortion of melanocytes or complete regression of the lesion.

According to the recommendations for photoepilation from the European Society for Laser Dermatology (ESLD), pigmented nevi should be avoided, or they should be covered with white adhesive tape” [47]. In many countries, a white kajal pencil that contains titanium dioxide is commonly used; however, the amount used seems insufficient to protect nevi [48] and clinical and dermoscopic changes in nevi have been observed despite its use [28]. Bodendorf et al. tested several materials in shielding nevi from accidental collateral effects during scanned laser epilation. In an in vitro absorption assay, transmission in the diode and alexandrite laser was reduced to 8.77% and 7.99%, respectively, after zinc oxide paste (1 g/cm^2^) application, to 8.05% and 3.62%, respectively, with a wooden spatula slide, to 19.85% and 16.91%, respectively, after sunscreen use, to 19.25% and 20.78%, respectively, after polyurethane foam, and to 76.43% and 71.03% after white kajal [48]. It was noted that wooden spatulas are not recommended for shielding nevi because of the danger of catching fire with repeated laser application [48].

In conclusion, clinical and dermoscopic changes and atypical findings have been observed in melanocytic lesions after photoepilation that prompted the further evaluation of the lesion with short-term sequential digital dermoscopy or surgical excision in order to establish an unequivocal diagnosis. These diagnostic approaches can be time-consuming, require familiarization of the physician with dermoscopic features, may cause anxiety to the individual, and highlight that avoiding passes of the laser or IPL devices over pigmented lesions is key.

## Figures and Tables

**Table 1:** Dermoscopic findings in pigmented lesions after laser or IPL hair removal.

Publication	Study Type	N Persons, *n* of Evaluated Nevi	Hair Removal Device	Dermoscopic Changes in Nevi after Laser or IPL Hair Removal
Acle, 2022 [18]	Prospective, blinded, and controlled	34 women, 148 nevi on the legs after photo-epilation. Arm nevi used as controls. Assessed at baseline and at sessions 2, 3, and 6 of photo-epilation	Diode	Before session 6: Any dermoscopic change in 47.9% of nevi (versus 9.8% in control nevi, *p* < 0.001) Most nevi maintained their original dermoscopic pattern. Bleaching: 41.9% (versus 0 in control nevi, *p* < 0.05). Pigmented globules in 6.7% (versus 0 in control nevi, *p* < 0.05). Irregular hyperpigmented areas in 5.4% (versus 0 in control nevi, *p*: 0.011). Regression structures in 4.7% (versus 0 in control nevi, *p*: 0.021).
Nasimi, 2021 [27]	Prospective	27 women, 66 nevi	Alexandrite	Changes in pigment network pattern in 93%. Changes in color in 64% (lighter in 44%, darker in 20%). Changes in dots/globules in 38%. Asymmetry in 12%. Regression in 3%.
Guicciardi, 2018 [19]	Prospective	18 persons, 73 nevi	Nd:Yag, IPL	Bleaching in 82%. Irregular pigment network at the periphery in 79%. New blue-grayish globules in 46.5%. New whitish structureless areas in 37%. Complete regression in 32.8%. Telangiectasias in 11%. Crusting in 11%. Progressive growth, peripheral globules in 1 nevus (0.13%).
Garrido-Rios, 2013 [14], Alvarez-Garrido, 2016 [28]	Case reports	1 person, 2 nevi	Diode	Loss of pigment network. New grayish areas.
		1 person, 2 nevi	Alexandrite	Loss of pigment network.
		1	IPL	Crusts.
Alvarez-Garrido, 2016 [28]	Case series	1 person, several nevi	Diode	Loss of pigment network, grayish areas.
		1 person, 1 nevus	IPL	Light pigment network, grayish areas.
		1 person, 1 nevus	Alexandrite	New grayish areas.
Sillard, 2013 [31]	Case reports	1 person, several nevi	IPL	Asymmetric pigment network. Gray-blue dots. Milky red veil.
		1 person, 1 nevus	Alexandrite	Asymmetric pigment network. Gray-blue dots. Milky red veil.
Martin, 2012 [29]	Case report	1 person, 1 nevus	IPL	Complete loss of the pre-existing reticular pattern. New blotches of brown pigment. Whitish areas, centrally located.
Boleira [21]	Case report	1 person, 1 nevus	Diode	Homogeneous featureless pattern.
Pampin Franco [30]	Caser reports	1 person, 1 nevus	IPL	Eccentric reticular pattern. Blue-grayish area in over 80% of the lesion. Different from the person’s signature pattern nevus.
		1 person, several nevi	Laser	Blue-grayish-whitish areas on several nevi.

**Table 2:** Diagnostic approach to dermoscopic findings and changes in pigmented lesions observed after laser or IPL hair removal.

Author	Reason for Concern	Diagnostic Approach
Alvarez-Garrido [28]	Dermoscopic change in one nevus with sequential digital dermoscopy. New gray areas in a nevus that previously had a homogenous brown pattern. Family history of melanoma in her mother	Excision
	Dermoscopic changes in several nevi with sequential digital dermoscopy. Loss of pigment network, new grayish areas.	Sequential digital dermoscopy follow-up that showed persistent findings in most lesions after 4 months
	Dermoscopic findings in one nevus. Grayish areas and a light pigment network.	Sequential digital dermoscopy follow-up that showed persistent findings after 6 months
	Referred by his general doctor for changes in several nevi. Grayish areas	Sequential digital dermoscopy follow-up that showed persistent findings after 6 months
Ashack [20]	Ugly duckling in a nevus that was darker and bigger than other nevi.	Excision
Boleira [21]	Ugly duckling of a nevus that was darker and different with dermoscopy from other nevi Family history of melanoma.	Excision
Pampin Franco [30]	Atypical nevus syndrome Blue-grayish-whitish areas in sequential digital dermoscopy	Reflectance confocal microscopy Excision
	Atypical nevus syndrome Blue-grayish-whitish areas in sequential digital dermoscopy	Reflectance confocal microscopy Excision
Soden [24]	History of dysplastic nevi Clinical changes with asymmetry, irregular borders, and color change	Biopsy
	Color change Family history of melanoma	Excision
Sillard [31]	Asymmetric pigment network with numerous gray-blue dots and a milky red veil.	Excision
Martin [29]	Family history of melanoma No melanocytic pattern. Central whitish areas	Short-term follow-up Excision
Garrido-Rios, 2013 [14]	Loss of pigment network. New grayish areas	Excision
	Reinforcement of the reticular pattern in several nevi. Personal and family history of melanoma.	Follow-up and resolution of changes after 3 months.

**Table 3:** Published cases with follow-up of nevi after they received passes with laser or IPL hair removal devices.

Publication	N Persons with Nevi Followed Up	Hair Removal Device	Follow-Up Time, Months	Outcome of Changes
Guicciardi, 2018 [19]	18 persons with 73 nevi	Nd:Yag, IPL	At least 24	Persisted, stable. One lesion showed progressive growth, had peripheral globules, and was excised. Histopathology showed a compound nevus with slight atypia
Acle, 2022 [18]	34 women with 148 nevi on the legs	Diode	4–6 (6 sessions)	Persisted. Bleaching in 41.9% of nevi. Pigmented globules in 6.7%. Irregular hyperpigmented areas in 5.4%. Regression structures in 4.7%
Garrido-Rios, 2013 [14], Alvarez-Garrido, 2016 [28]	1	Diode	36	Persisted
	1	Alexandrite	36	Persisted
	1	IPL	3	Resolved
Alvarez-Garrido, 2016 [28]	1	Diode	4	Persisted in most nevi despite stopping photoepilation
	1	IPL	6	Persisted despite stopping photoepilation
	1	Alexandrite	6	Persisted despite stopping photoepilation
Sillard, 2013 [31]	1	IPL	6	Resolved
Martin, 2012 [29]	1	IPL	3	Persisted. New brownish dots in diffuse distribution. Lesion excised and histopathology showed complete regression of the nevus

N: number, IPL: intense pulsed light.

**Table 4:** Histological findings in atypical pigmented lesions excised after laser or IPL hair removal.

Author	Reasons for Excision	Histological Findings	Diagnosis
Garrido-Rios [14]	Loss of pigment network. New grayish areas	Melanophages in the upper dermis. One compound nevus and one lentiginous nevus.	Nevi
	Loss of pigment network	Loss of pigment in the epidermo–dermal junction, especially near the hair follicles. Melanocytic compound nevi	Nevi
Guicciardi [19]	Progressive growth of one lesion with appearance of globules at the periphery.	Compound nevus with slight atypia	Nevus
Ashack [20]	Dark lesion, different from the person’s signature nevus	Biopsy of the lesion showed epidermal and superficial dermal changes in electrical and thermal injury, with adjacent junctional melanocytic proliferation with follicular extension. A surgical excision was performed and histology showed a crusted gray-pink scar. The melanocytes were distorted histologically	No diagnosis
Boleira [21]	Ugly duckling Featureless pattern Family history of melanoma.	Scale crust permeated by melanin and numerous melanophages. Triangular basophilic collagen degeneration with the vertex pointed down in the papillary dermis, characteristic of laser injury. No evidence of a residual melanocytic lesion	Regressed melanocytic lesion. No diagnosis
Soden [24]	Color change Family history of melanoma	Subepidermal blister. Melanocytes with marked distortion in their shape or fragmented within the epidermis or the dermal–epidermal junction. There was collagen homogenization in the papillary dermis and small foci of residual nevus cells in the dermis.	Nevus
Alvarez-Garrido [28]	New grayish areas in a nevus that previously had a homogenous brown pattern. Family history of melanoma.	Lentiginous nevus with an increased number of melanophages in the upper dermis.	Nevus
Martin [29]	Complete loss of the pre-existing reticular pattern. New blotches of crusted brown pigment. Whitish areas, centrally located.	Complete regression of the pre-existing melanocytic nevus. Superficial microcrusts. The papillary dermis showed fibrosis and melanophages.	No diagnosis
Pampin Franco [30]	Eccentric reticular pattern Blue-grayish area in over 80% of the lesion. Different from the person’s signature pattern nevus Atypical nevus syndrome	Edema, fibrosis, capillary neoformation, pigment incontinence, and melanophages in papillary dermis. Isolated nevocellular nests in the dermo–epidermal junction.	Nevus
	Clearance of nevus Blue-grayish-whitish areas Atypical nevus syndrome	Compound nevus with architectural distortion and focal regression without cellular atypia.	Nevus
Sillard [31]	Asymmetric pigment network, numerous gray-blue dots, and a milky red veil.	Compound nevus with melanophages.	Nevus
	Personal history of melanoma Change with asymmetric pigment network, gray-blue dots, and a milky red veil.	Melanocytic junctional proliferation with mild atypia. Increase in vascularization, melanophages, and dermal fibrosis.	Nevus

## Data Availability

Not applicable.
